# Application of the E-value Under Nonproportional Hazards

**DOI:** 10.1097/EDE.0000000000002017

**Published:** 2026-07-06

**Authors:** Carmen A.T. Reep, Evert-Jan Wils, Leo Heunks

**Affiliations:** 1 Department of Intensive Care, Erasmus Medical Center, Rotterdam, the Netherlands; 2 Department of Intensive Care, Radboud University Medical Center, Nijmegen, the Netherlands, c.reep@erasmusmc.nl; 3 Department of Intensive Care, Franciscus Gasthuis & Vlietland, Rotterdam, the Netherlands; 4 Department of Intensive Care, Radboud University Medical Center, Nijmegen, the Netherlands

## To the Editor:

The E-value provides a robust metric for assessing the potential impact of unmeasured confounding in observational data studies aimed at causal inference.^1^ It represents the minimum strength of association, on the risk ratio scale, that an unmeasured confounder must have with both treatment and outcome to fully explain away a specific treatment-outcome association, conditional on the measured covariates.^1^ Larger E-values indicate stronger confounding is needed to negate the association.^1^ Standard applications of the E-value typically report a single, static measure at the end of follow-up. However, this creates a methodological gap in settings where the hazard ratio does not remain constant from the first day of follow-up through the end of the study—a scenario typical of most medical interventions.^2^ This limitation is particularly evident when interest lies in how effects evolve over time.

For example, in critically ill patients on invasive mechanical ventilation, switching from controlled to assisted ventilation is the first step toward extubation. A key question is whether earlier switching leads to earlier or more frequent successful extubation,^3^ thereby potentially reducing complications and Intensive Care Unit resource use. Because all survivors are eventually extubated, cumulative incidences converge over time, making end-of-follow-up risk differences, ratios, and corresponding E-values less informative. Alternative measures, such as the restricted mean time lost, which captures the area under the cumulative incidence curve,^4^ may be more informative. However, the framework by VanderWeele and colleagues does not address E-value reporting in this setting. We therefore propose guidance for reporting and interpreting E-values when proportional hazards do not hold, and the goal is to understand how effects evolve over time.

One approach is to report the restricted mean time lost effect estimate for the entire follow-up period alongside risk estimates and corresponding E-values for a small number of clinically meaningful time points. E-values are calculated using the point estimate and the confidence limit closest to the null at each time point, noting that the E-value can flip directions when effect directions differ over time.^1^

**FIGURE 1.**
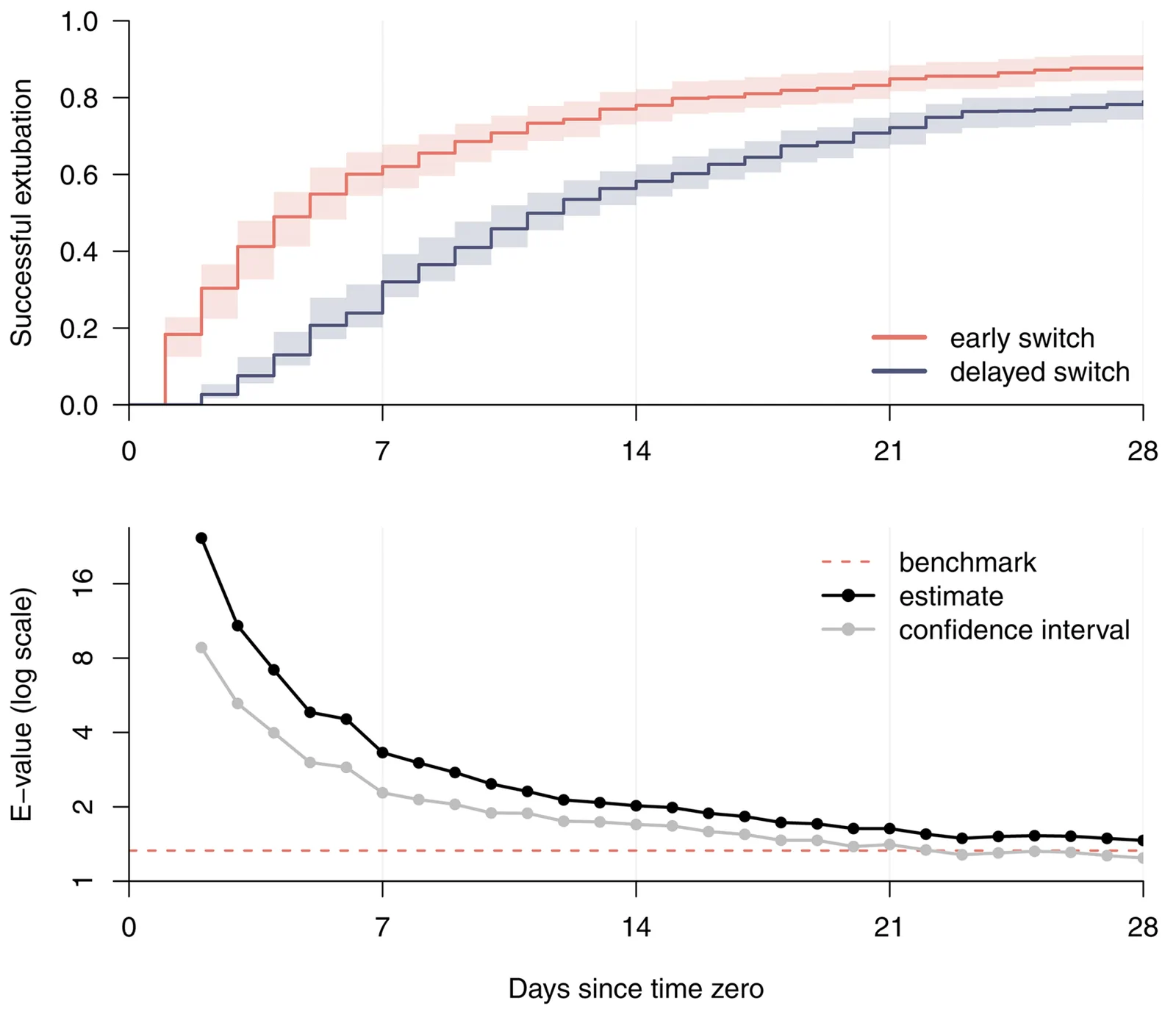
E-Value for ventilation mode switching under conditions of nonproportional hazards. The analysis compares an early switch strategy (within 1 day after meeting eligibility; n = 469) against a delayed switch strategy (after 1 day; n =507). Eligibility was defined as ≥2 days on controlled invasive mechanical ventilation, absence of neuromuscular blockers, and a PaO_2_/FiO_2_ ratio >150 mm Hg. At each time point, E-values were calculated using both the point estimate and the confidence limit closest to the null of the relative risk (i.e., 1) between treatment arms. The larger the E-value, the stronger the confounding associations must be to explain away the effect estimate. The benchmark line represents the baseline hazard ratio (HR) of the strongest measured confounder for the outcome successful extubation (sedation level [awake; moderate; deep], HR = 1.38). This benchmark is provided for context only and should not be interpreted as a decision threshold. E-values substantially exceeding this benchmark suggest that it is unlikely unmeasured confounding alone could fully explain the observed effect. Top panel adapted from Figure 2A in Reep et al., Am J Respir Crit Care Med 2025, with permission from Oxford University Press.

Alternatively, E-values can be calculated and plotted over the full follow-up. This longitudinal approach avoids selection of arbitrary time points and yields a continuous sensitivity profile aligned with cumulative incidence curves. We illustrate this using the switch example with MIMIC-IV data^5^ (Figure 1), targeting the total effect on successful extubation while treating death as a competing event without elimination.^6^ As a context-specific benchmark for confounding, we compared results to the strongest measured confounder at the time of meeting eligibility criteria: sedation level (awake, moderate, and deep). The hazard ratio was 0.72 (inverse 1.38), indicating higher baseline sedation is associated with delayed extubation. Although this baseline estimate is a pragmatic simplification of the time-varying nature of confounding and the “at-risk” set, it provides a pragmatic reference. In our example, a single unmeasured confounder stronger than sedation is highly unlikely. Because multiple weaker confounders acting in the same direction could jointly generate bias,^7^ it is also necessary to consider whether a cluster of unmeasured factors could collectively rival this strength. In our analysis, E-values (especially early in follow-up) are substantially larger than the effect of sedation level, suggesting that even combined unmeasured confounding is unlikely to explain the observed effect.

To conclude, for observational studies aiming to estimate causal effects, where hazards are not proportional over time, reporting the E-value only at the end of follow-up may be misleading. Evaluating E-values across multiple time points provides a more informative assessment of the potential impact of unmeasured confounding. Comparing these E-values with the strongest measured confounder as a context-specific benchmark for the magnitude of confounding and incorporating expert judgment further strengthens confidence in the results.
